# Transplantation of bFGF-expressing neural stem cells promotes cell migration and functional recovery in rat brain after transient ischemic stroke

**DOI:** 10.18632/oncotarget.22155

**Published:** 2017-10-27

**Authors:** Jin-Jing Zhang, Juan-Juan Zhu, Yuan-Bo Hu, Guang-Heng Xiang, Lian-Cheng Deng, Fen-Zan Wu, Xiao-Jie Wei, Ying-Hao Wang, Liang-Yan Sun, Xiao-Qing Lou, Min-Min Shao, Mao Mao, Hong-Yu Zhang, Yue-Ping Xu, Si-Pin Zhu, Jian Xiao

**Affiliations:** ^1^ Department of Pharmacy, Affiliated Cixi People's Hospital, Wenzhou Medical University, Ningbo, Zhejiang, 315300, China; ^2^ Department of Orthopaedics, The Second Affiliated Hospital and Yuying Children's Hospital of Wenzhou Medical University, Wenzhou, Zhejiang, 325027, China; ^3^ Department of Geriatrics and Neurology, The Second Affiliated Hospital and Yuying Children's Hospital of Wenzhou Medical University, Wenzhou, Zhejiang, 325027, China; ^4^ Institute of Molecular Pharmacology, School of Pharmaceutics Science, Wenzhou Medical University, Wenzhou, Zhejiang, 325035, China; ^5^ Department of Neurosurgery, Affiliated Cixi People's Hospital, Wenzhou Medical University, Ningbo, Zhejiang, 315300, China

**Keywords:** aging, neural stem cells, basic fibroblast growth factor, ischemic stroke, cell therapy

## Abstract

Cerebrovascular disease such as stroke is one of the most common diseases in the aging population, and neural stem cells (NSCs) transplantation may provide an alternative therapy for cerebral ischemia. However, a hostile microenvironment in the ischemic brain offers is challenging for the survival of the transplanted cells. Considering the neuroprotective role of basic fibroblast growth factor (bFGF), the present study investigated whether bFGF gene-modified NSCs could improve the neurological function deficit after transient middle cerebral artery occlusion (MCAO) in adult male Sprague–Dawley rats. These rats were intravenously injected with modified NSCs (5×10^6^/200 μL) or vehicle 24 h after MCAO. Histological analysis was performed on days 7 and 28 after tMCAO. The survival, migration, proliferation, and differentiation of the transplanted modified C17.2 cells in the brain were improved. In addition, the intravenous infusion of NSCs and *bFGF* gene-modified C17.2 cells improved the functional recovery as compared to the control. Furthermore, bFGF promoted the C17.2 cell growth, survival, and differentiation into mature neurons within the infarct region. These data suggested that bFGF gene-modified NSCs have the potential to be a therapeutic agent in brain ischemia.

## INTRODUCTION

Aging is a major risk factor of disease-susceptible conditions and deaths around the world, and cerebrovascular disease is one of the leading causes of morbidity and mortality in elderly people. The cerebrovascular disease is a consequence of impaired blood supply in the brain; thus, ischemic events account for 80% of all strokes [1]. However, the current treatment of ischemic stroke remains an intimidating mission as only a few therapeutic strategies have been proven to be effective. Systemic thrombolysis is the only validated therapy that improves the clinical outcome; nevertheless, not all patients can receive this therapy as it must be administered within 4.5 h after onset [2, 3]. Therefore, the development of alternative therapies for ischemic stroke is essential.

Cell therapy is emerging as a viable neurorestorative therapy for stroke [4]. The neural stem cells (NSCs) constitute the most attractive alternative owing to the regenerative capacity and minimal immunogenic characteristic. NSCs persist in the subventricular zone (SVZ) of the lateral ventricle and the subgranular zone (SGZ) of the dentate gyrus throughout the adulthood in mammals. NSCs can differentiate into neurons, astrocytes, oligodendrocytes, and endothelium cells, which constitute the majority of the cerebral cell types affected by ischemic insult [5–7]. Therefore, transplantation of NSCs is a potential treatment for stroke [8]. However, a hostile microenvironment in the ischemic regions is challenging for the survival of transplanted cells as only a few grafted cells survive for a prolonged duration [9]. Therefore, improving the survival of the grafted stem cells and inducing their differentiation into cerebral cell types are issues of paramount importance.

Basic fibroblast growth factor (bFGF) is a critical neurotrophic factor that can improve the sensorimotor recovery after stroke [10, 11]. It can accelerate the key processes of the cell, such as survival, proliferation, and differentiation [12–14]. This neurotrophic factor, produced by astrocytes, is lowly expressed in the adult mammalian brain tissue. However, the level of bFGF is significantly upregulated as a consequence of brain injury [15]. Several groups have found that exogenous neurotrophic factors may play a crucial role in resistance to ischemic injury [16, 17], and bFGF has been demonstrated to enhance the proliferation and differentiation of endogenous neural progenitor cells [18, 19]. The intracisternal administration of bFGF at the beginning of 24 h post-stroke promoted recovery and stimulated the sprouting of new neurons as well as synapse formation [20].

In the present study, we tested whether intravenously injected *bFGF* gene-modified NSCs could improve the neurological functional recovery and reduction of cerebral infarction volume after focal stroke in rats. In addition, we determined the survival, migration, and proliferation abilities of *bFGF* gene-modified NSCs in the ischemic brain microenvironment.

## RESULTS

### bFGF promotes the survival of the C17.2 cell after oxygen-glucose deprivation (OGD)

**Figure 1:**
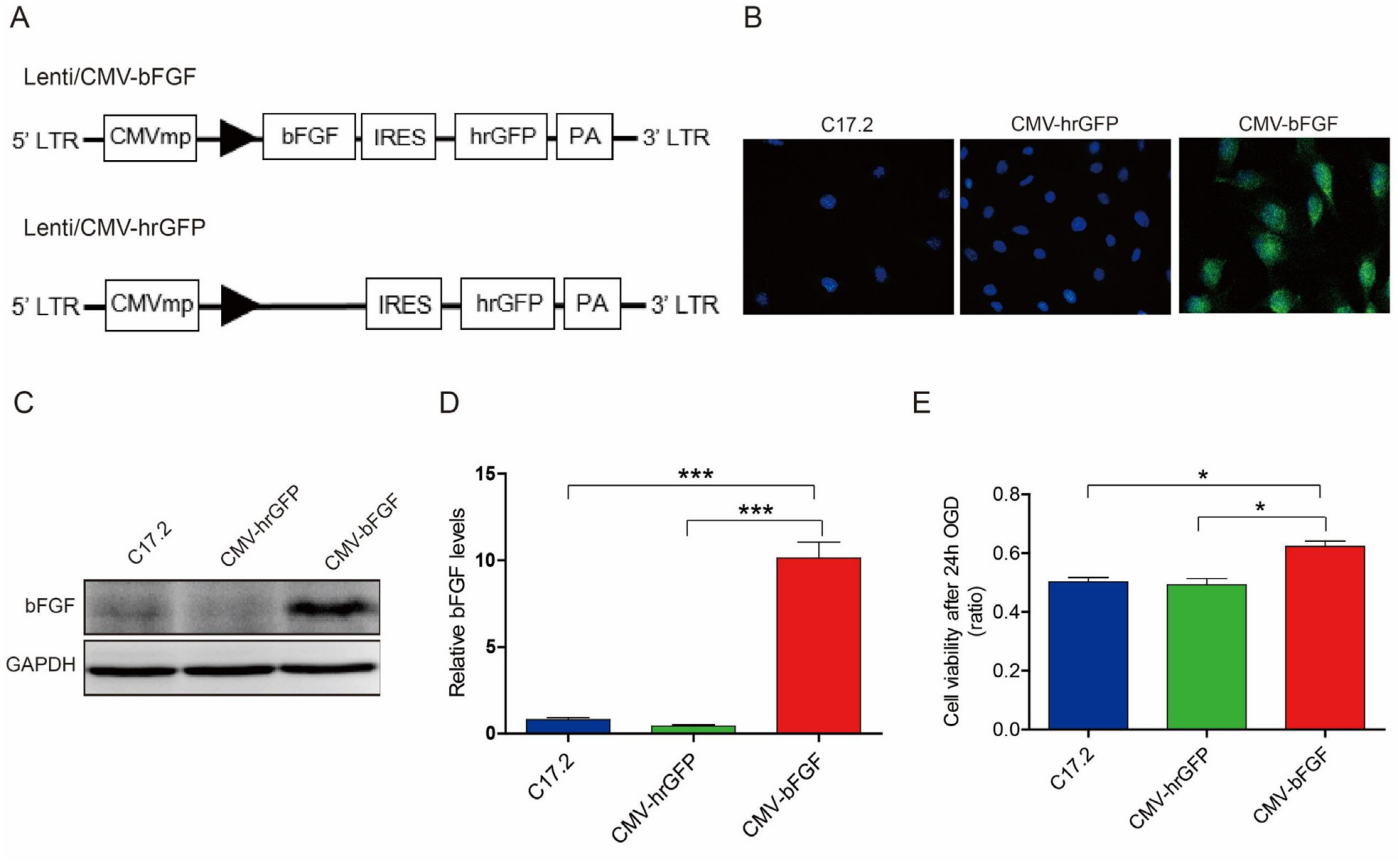
The expression of bFGF *in vitro* and survival of NSCs after OGD **(A)** The schematic of the two vectors. **(B, C, D)** Immunofluorescence and Western blot analysis of bFGF expression in CMV-bFGF C17.2, CMV-hrGFP C17.2, and C17.2 cells. The level of bFGF is significantly upregulated in CMV-bFGF C17.2 cells. The error bars represent the means ± SEM of three independent experiments; ^***^P < 0.001. **(E)** The cell viability in OGD was detected by MTT assay, and bFGF significantly enhanced the cell viability under OGD. The error bars represent the means ± SEM of three independent experiments; ^*^P < 0.05.

bFGF plays a major role in the development of nervous system and injury repair [21]. Therefore, we established the highly expressing *bFGF* gene-modified neural stem cells, and the hrGFP construct was transfected into the cells to be used as control (Figure 1A). Immunofluorescence and Western blot showed greater bFGF protein expression in CMV-bFGF C17.2 cells as compared to the CMV-hrGFP C17.2 and uninfected C17.2 cells (Figure 1B–1D).

OGD was used to simulate the environment of cerebral ischemia. As shown in Figure 1E, the viability of the cells was increased significantly in the CMV-bFGF C17.2 cells as compared to the CMV-hrGFP C17.2 and C17.2 cells (*P* < 0.05) after 24 h OGD. Taken together, these results suggested that CMV-bFGF C17.2 had a greater proliferative ability, and bFGF promotes cells survival under OGD.

### Administration of CMV-bFGF C17.2 cells improves the functional recovery after middle cerebral artery occlusion (MCAO)

**Figure 2:**
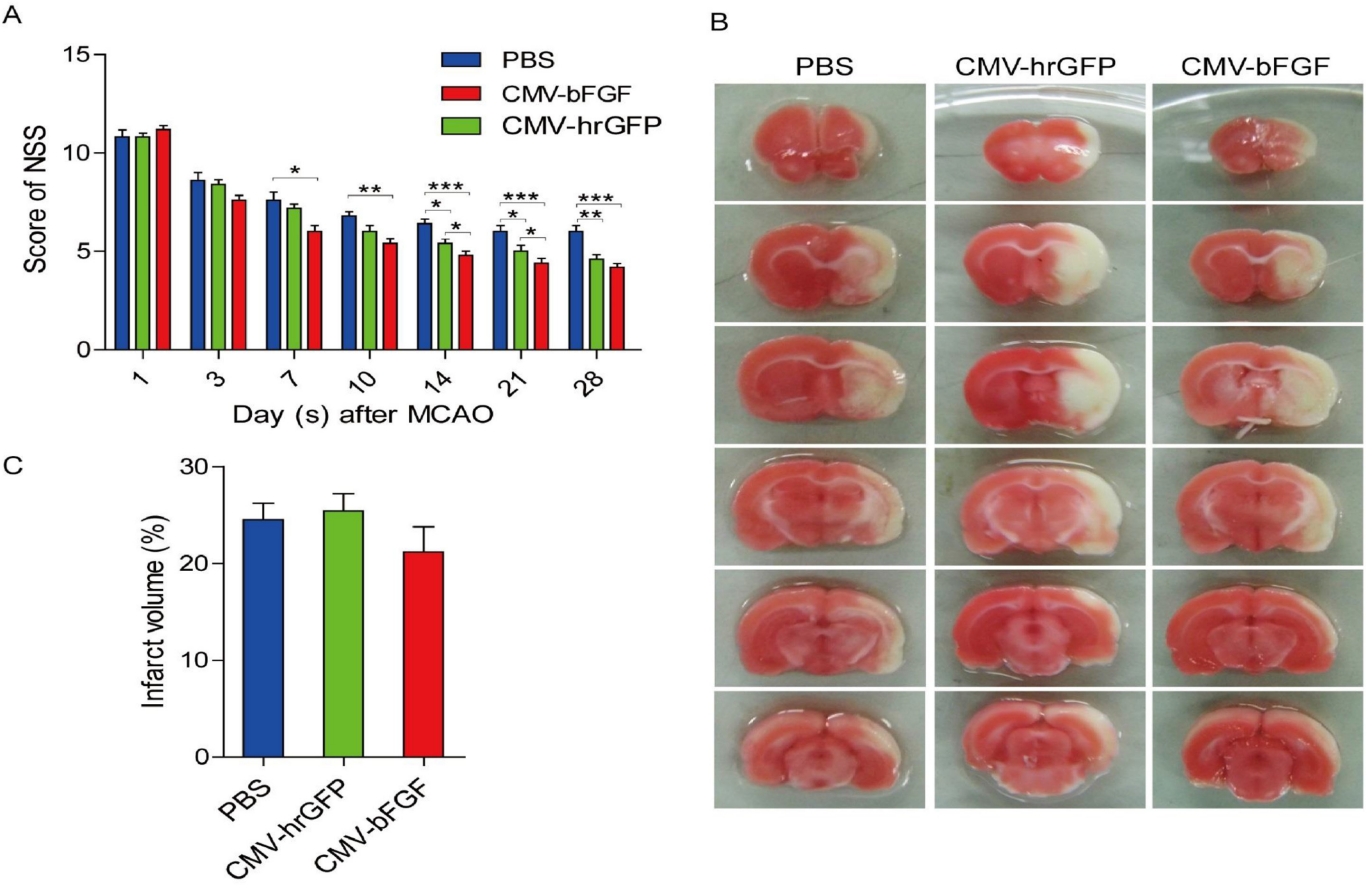
Effect of intravenously transplanted NSCs on neurological function deficit and cerebral infarction volume in ischemic stroke rats **(A)** Behavioral performance in the NSS of CMV-bFGF C17.2-, CMV-hrGFP C17.2-, and PBS-treated groups from days 1–28 after ischemia (n = 6, each group). The functional assessment revealed a significant improvement in NSS at 14 days post-MCAO in CMV-bFGF C17.2- and CMV-hrGFP C17.2-treated rats. **(B)** Brain slices were stained with TTC to visualize lesions (n = 5, each group). **(C)** The infarction volume was calculated by Image J software and results summarized. No significant differences in the infarct volume in the CMV-bFGF C17.2 group as compared to the CMV-hrGFP C17.2 and PBS groups. The error bars represent the means ± SEM; ^*^P < 0.05, ^**^P < 0.01, ^***^P < 0.001.

The neurological severity scores (NSS) were calculated based on a series of motor sensory, reflex, and balance tests [22]. We used the NSS test to investigate whether CMV-bFGF C17.2 cells exhibited a better therapeutic effect than the unmodified NSCs after stroke. As evidenced by improved NSS scores, treatment with intravenously injected CMV-bFGF C17.2 cells 24 h post-MCAO significantly improved the functional recovery (Figure 2A). The evaluation of the function revealed a remarkable advance in NSS at 7 days post-MCAO in CMV-bFGF C17.2 cells and 14 days post-MCAO in CMV-hrGFP C17.2 cells. These results demonstrated that the functional deficits resulting from transient focal cerebral ischemia in rats effectuate a remarkable improvement by intravenous transplantation of CMV-bFGF C17.2 cells.

We compared the infarction areas in coronal sections from animals of the PBS, CMV-bFGF C17.2 and CMV-hrGFP C17.2 groups on day 7 (Figure 2B). The normal brain tissue typically stained with 2, 3, 5-triphenyltetrazolium chloride (TTC); however, the infarcted lesions showed limited or no staining. The TTC staining was used to assess the lesion volume as a percentage of contralateral hemispheric volume. However, no significant differences were detected in the infarct volume in the CMV-bFGF C17.2 group as compared to the CMV-hrGFP C17.2 and PBS groups (Figure 2C).

### bFGF promotes NSCs migration into ischemic brain and increases survival

**Figure 3:**
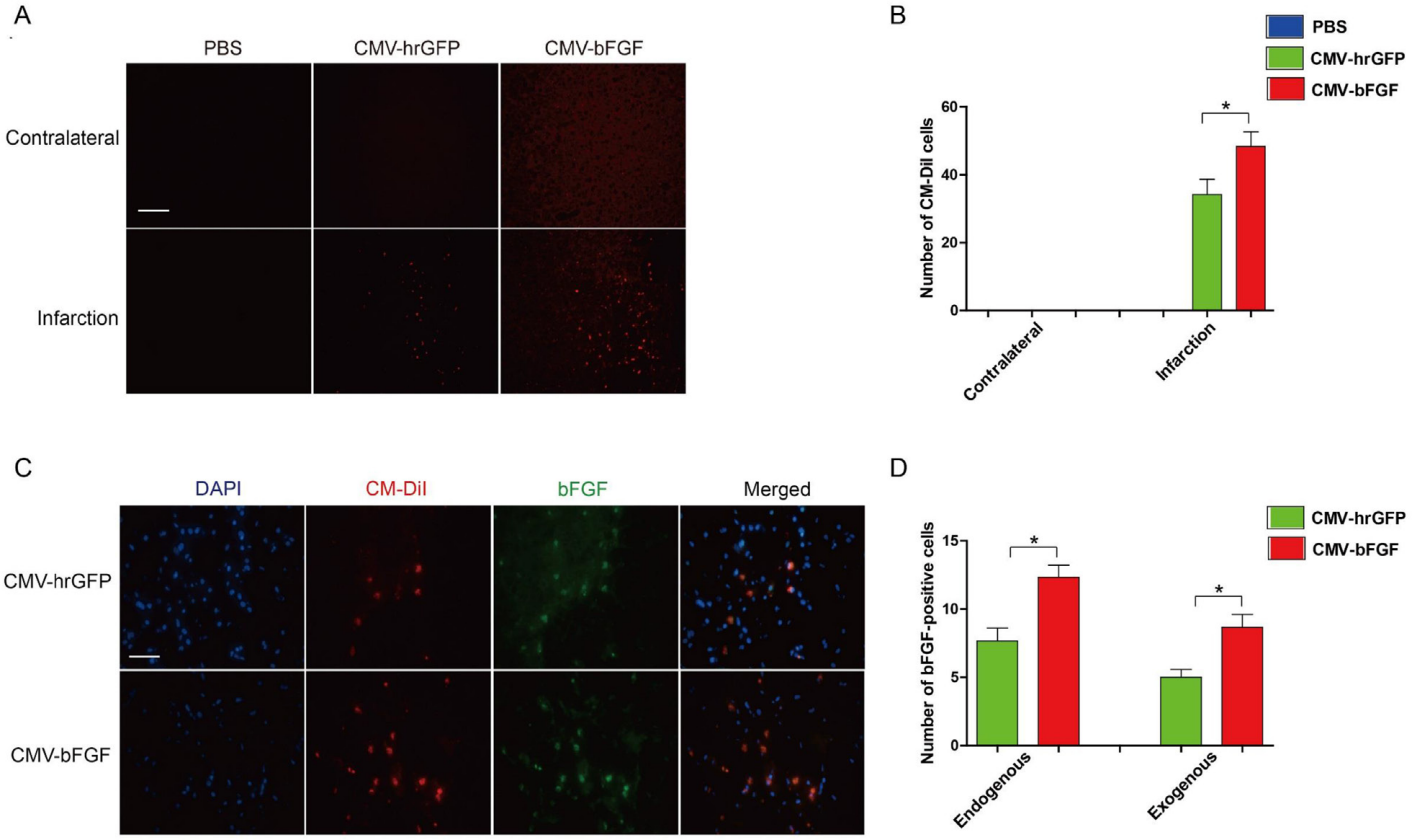
Effects of intravenously transplanted NSCs on migration and survival in ischemic stroke rats **(A)** The distribution of transplanted NSCs in the infarct region. Cell Tacker CM-DiI-positive cells were shown in red. The cells were mainly limited to the infarcted areas, although some were sparsely observed throughout the affected hemisphere (Scale bar: 200 μm). **(B)** The number of CM-DiI-positive cells was measured in the contralateral and infarction. **(C)** The expression of bFGF in the infarct region at 28 days after MCAO. bFGF promotes NSCs’ migration and maintains the survival of cells in the infarcted lesion. CM-DiI-positive cells were shown in red; bFGF-immunopositive cells were shown in green (scale bar: 50 μm). **(D)** The number of bFGF-positive cells was measured in the infarct area. CM-DiI and bFGF-positive co-labeled cells representing the exogenous NSCs; bFGF-positive single cells representing the endogenous cells. The error bars represent the means ± SEM, n = 6 each group; ^*^P < 0.05.

To confirm whether the CMV-bFGF C17.2 cells effectuated greater functional recovery, all cells were pre-labeled with red fluorescent dye CM-DiI before transplantation. As shown in Figure 3A and 3B, transplanted NSCs were widely distributed throughout the ipsilateral cerebral hemisphere; however, they were not detectable in the contralateral hemisphere. Also, a large number of CMV-bFGF C17.2 cells were found in the ischemic region than the CMV-hrGFP C17.2 cells. Additionally, the expression of bFGF was examined in the infarcted lesion. As shown in Figure 3C and 3D, the CMV-bFGF C17.2 group had more bFGF-positive cells than the CMV-hrGFP C17.2 group at 28 days after MCAO. Thus, the animals treated with CMV-bFGF C17.2 cells after ischemia displayed an increased rate of migration and survival of NSCs, suggesting that bFGF promotes the migration of NSCs into the ischemic brain and maintains the survival of cells in the infarcted lesion.

### bFGF-modified NSCs maintain proliferative capability

**Figure 4:**
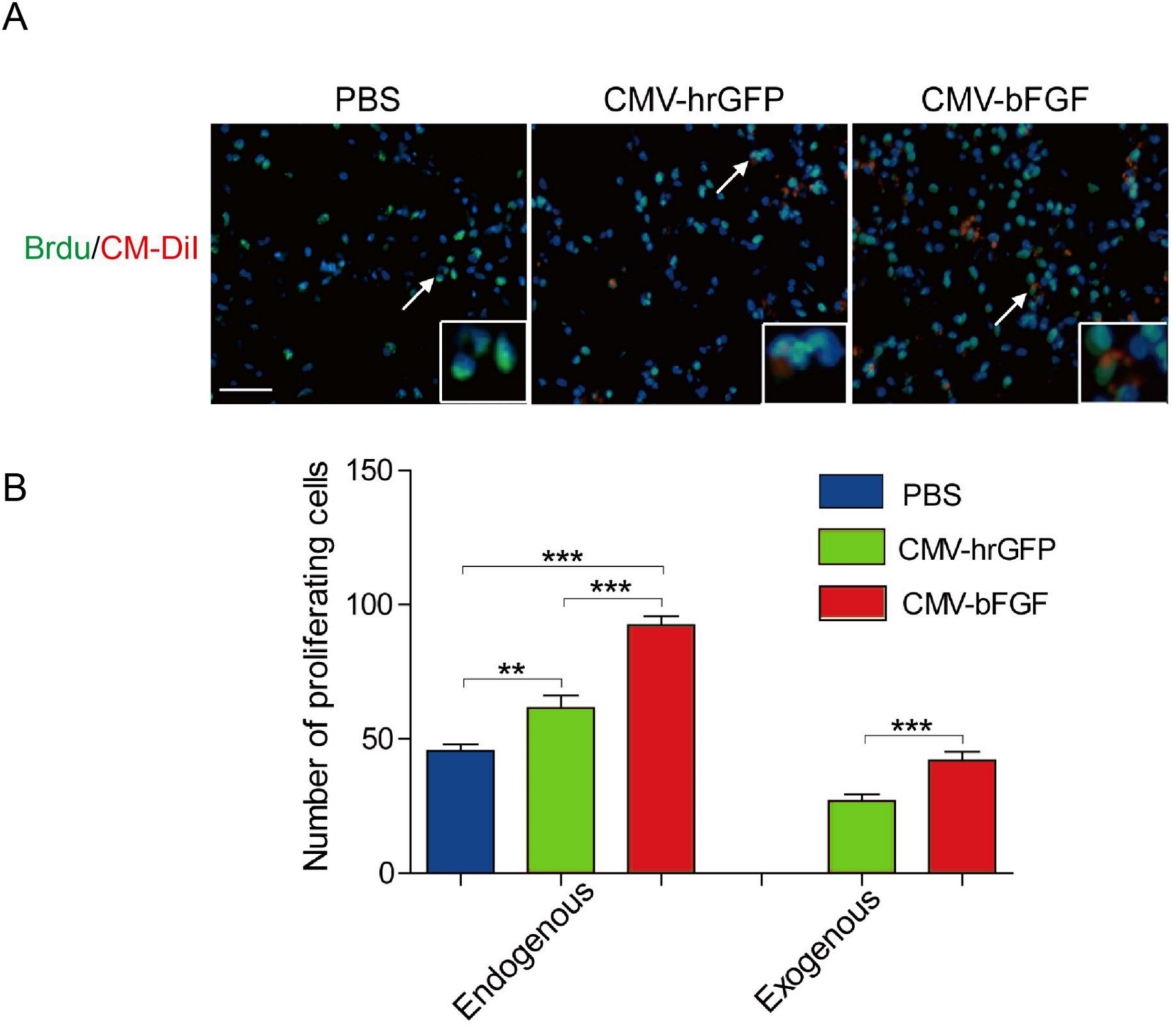
Effect of intravenously transplanted NSCs on proliferation in ischemic stroke rats **(A)** Representative images of exogenous and endogenous by BrdU-labeled cells were shown. CM-DiI-positive cells were shown in red; BrdU-positive cells were shown in green. **(B)** The number of BrdU-positive cells was estimated in the infarct area. bFGF promotes NSCs’ proliferation *in vivo*. CM-DiI and BrdU-positive co-labeled cells represented the exogenous NSCs; BrdU-positive single cells represented the endogenous cells. The error bars represent the means ± SEM, n = 5 each group; ^**^P < 0.01, ^***^P < 0.001 (scale bar: 50 μm).

The proliferation ability of transplanted NSCs in the ischemic region was analyzed by 5-bromo-2’-deoxyuridine (BrdU), a thymidine analog that is incorporated into DNA during cell division. As shown in Figure 4A, a large number of BrdU-positive cells were observed in ischemic hemispheres of two NSCs-treated rats as compared to the PBS-treated control. Approximately, 45% of the transplanted CMV-bFGF C17.2 cells and 30% of the transplanted CMV-hrGFP C17.2 cells were overlapped with BrdU staining, indicating that a majority of them continued to proliferate in the ischemic environment. Moreover, the CMV-bFGF C17.2 cells had a greater proliferation ability at least 28 days after MCAO.

Notably, most of the BrdU-positive cells were not co-localized with CM-DiI in NSCs-treated rats, suggesting that they were proliferating endogenous cells (Figure 4B). A significant increase was observed in the proliferating endogenous cells in NSCs-treated ischemic regions as compared to the PBS-treated control. Taken together, these results demonstrated that transplanted NSCs could not only maintain their proliferative capacity within a hostile environment *in vivo* but also enhanced the proliferation of endogenous cells.

### bFGF promotes NSCs differentiation into neurons and astrocytes *in vivo*

**Figure 5:**
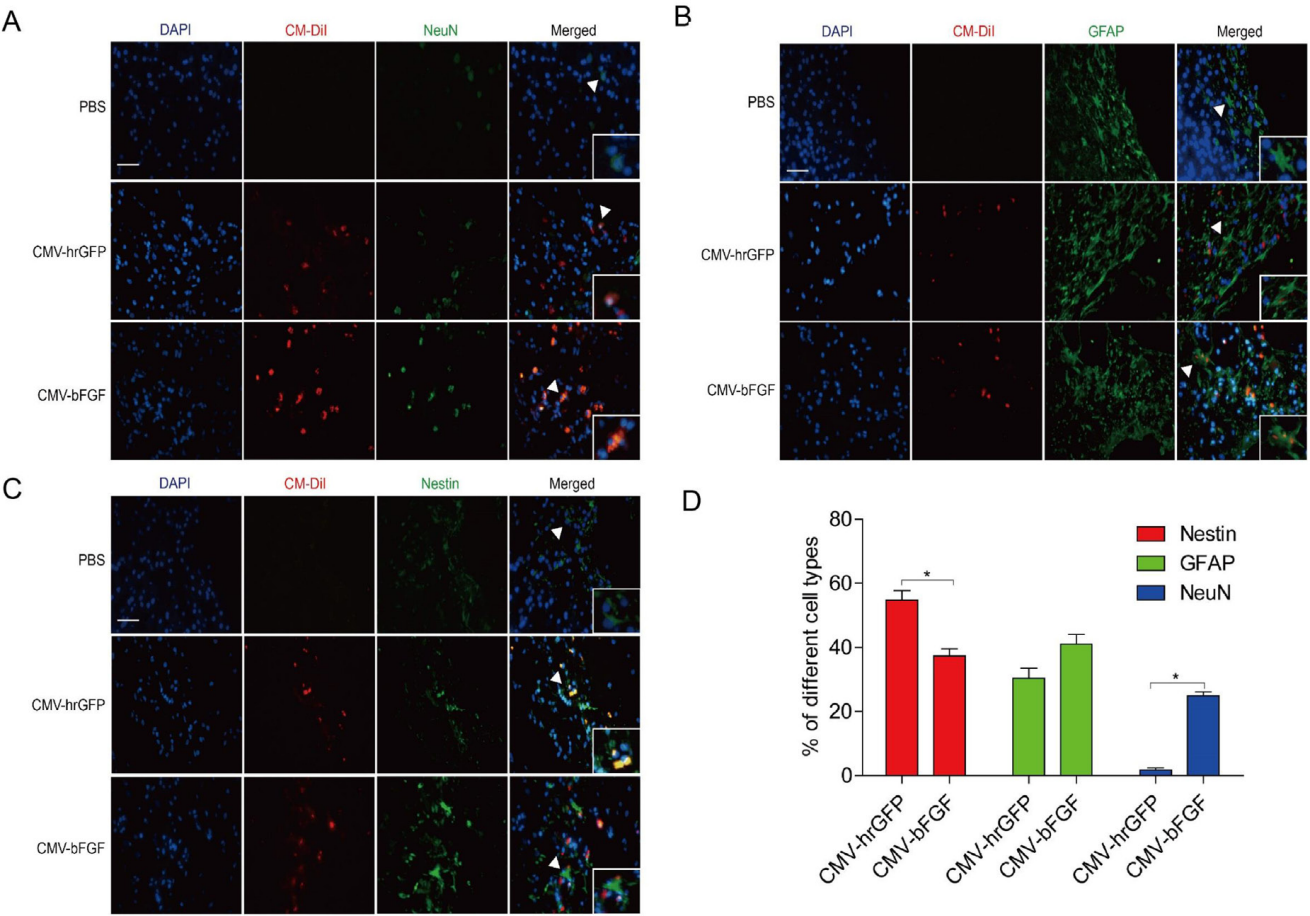
Effect of intravenously transplanted NSCs differentiation in ischemic stroke rats Immunofluorescent studies were carried out to identify NeuN **(A)**, GFAP **(B)**, and Nestin **(C)** in the infarct region in rats transplanted with CMV-bFGF C17.2, CMV-hrGFP C17.2, and PBS at 28 days after MCAO. Nestin, GFAP, and NeuN were co-localized with Cell Tracker CM-DiI, respectively. **(D)** Quantification of the differentiation of NSCs stained with CM-DiI in the infarct area. bFGF promoted differentiation of NSCs into mature neurons in the infarcted lesion. The error bars represent the means ± SEM, n = 6 each group; ^*^P < 0.05 (scale bar: 50 μm).

Immunofluorescent studies were carried out to identify NeuN (mature neurons), GFAP (astrocytes), and Nestin (a neural stem cell marker) in the infarcted lesion of rats at 28 days after MCAO (Figure 5). As shown in Figure 5A, transplantation with either CMV-bFGF C17.2 cells or CMV-hrGFP C17.2 cells significantly increased the number of NeuN-positive cells in the lesion zone. Furthermore, CMV-bFGF C17.2 cells exhibited an additional effect that increased the NeuN-positive cells co-localized with CM-DiI as compared to the CMV-hrGFP C17.2 cells, whereas the number of GFAP immunoreactive cells co-localized with CM-DiI cells showed no significant differences between the two groups (Figure 5B). Interestingly, the number of Nestin co-localized with CM-DiI cells in the CMV-bFGF C17.2 group was significantly less than that in the CMV-hrGFP C17.2 group (Figure 5C). Additionally, the NeuN-positive mature neurons, GFAP-positive astrocytes, and Nestin-positive NSCs were calculated, indicating that the transplanted cells mainly differentiated into NeuN in the ischemic area (Figure 5D). These results demonstrated that bFGF promotes differentiation of NSCs into mature neurons and astrocytes in the infarcted lesion.

## DISCUSSION

In the present study, we demonstrated that transplantation of bFGF-expressing NSCs is an efficient tool to improve the functional recovery in the rat model of focal stroke. However, the improvement of the neurological function was not accompanied by a reduction in the infarct volume as detected by TTC staining. This phenomenon might be attributed to the functional recovery that occurred solely without histological recovery at this specified time point. The precise mechanism underlying the functional improvement by cell transplantation in the ischemic brain is unknown. Some studies reported that several million stem/progenitor cells exhibit a certain degree of targeted migration towards the damaged regions post-transplantation into stroke animals [23]. One of the mechanisms of functional improvement in neural transplantation is the replacement or augmentation of neural circuits by transplanted NSCs; the other is associated with the release of trophic factors from the transplanted cells [24]. The intravenously administered NSCs can follow the gradients of chemo-attractants, such as vascular cell adhesion molecule 1 (VCAM-1) and stromal-derived factor 1 (SDF-1), enter the rat brain, survive, and migrate to the infarct area [25]. In the rat models of focal stroke, several studies revealed that bFGF treatment could enhance the proliferation, migration, and differentiation of endogenous neural progenitor cells [26, 27]. Our results showed that neurological functions in the C17.2 and *bFGF*-gene modified C17.2 groups were significantly improved as compared to the PBS control group as evaluated by NSS score from days 14–28 after ischemia. In addition, greater effects in the CMV-bFGF C17.2 group were observed at 14 days and 21 days after ischemia as compared to the CMV-hrGFP C17.2 stem cell-treated control group. However, no significant differences were detected in the infarct volume in the CMV-bFGF C17.2 group as compared to the CMV-hrGFP C17.2 and PBS groups at 7 days. Some investigators found that a considerable portion of grafted cells maintains an undifferentiated phenotype proximal or distal to the lesion of the host tissue, where these they can directly release the growth and trophic factors or promote the release of such factors from host brain cells [28–30]. This feature could be ascribed to the fact that improvement in neurological function was not accompanied by a reduction of the infarct volume in rats.

NSCs offer an alternative approach to treat cerebral ischemia owing to distinct biological features [31]. Firstly, NSCs is the most attractive option as they are minimally immunogenic and do not express mature cellular antigens, such as major histocompatibility complex (MHC) class 1 and 2 molecules [32]. Secondly, NSCs have a self-renewal function [33, 34]. Thirdly, NSCs can differentiate into neurons, astrocytes, and oligodendrocytes, thereby participating in the repair of nerve tissue; also, they can differentiate into non-nerve cells through straddled germinal layer differentiation [5]. The enhanced trophic activities of NSCs by overexpression of related genes would especially be valuable in amplifying the efficacy of cell therapies in the treatment of stroke [35]. Several studies have shown that bFGF treatment could enhance the proliferation, migration, and differentiation of endogenous neural progenitor cells after MCAO [10]. Thus, NSCs are utilized with *bFGF* genes to increase the percentage of migrated neurons. Our results showed that the viability of the cells significantly increased in the CMV-bFGF C17.2 cells as compared to the CMV-hrGFP C17.2 and C17.2 cells after 24 h OGD *in vitro*. A large number of CMV-bFGF C17.2 cells were found in the ischemic region at 4 weeks after transplantation than the CMV-hrGFP C17.2 cells.

We used the murine-derived immortalized multipotent neural progenitor cell line C17.2 to investigate whether *bFGF* gene-modified NSCs could improve the neurological functional recovery after focal stroke in rats. The murine C17.2 clone is a prototypical, stable, and extensively characterized NSC line [36]. Importantly, as a stem cell line, C17.2 NSCs contribute to the development of the organism throughout the neuraxis and across developmental time periods, from fetus to adult. Moreover, the construction of recombinant lentiviral vector is complex and time-consuming as the primary cells are unstable and aging [34]. Therefore, in this study, a cell line was selected rather than the primary cells in this experiment.

Currently, the optimal delivery route for specific cell types following cerebral ischemic injury is unknown. Thus, the present study chose intravenous delivery of NSCs at 24 h after MCAO/R in rats, which coincided with the time of opening of the blood-brain barrier (BBB) following ischemic stroke [37]. The intravenous infusion results in the entrapment of the transplanted cells in the filtering organs, thereby delivering a less number of NSCs into the brain than the intra-arterial methods. On the other hand, the intra-arterial delivery bypasses the peripheral filtering organs, leading to higher cell engraftment to the brain and greater efficacy as compared to intravenous infusion [38, 39]. The intracerebral delivery may be the most suitable method for NSC transplantation that can implant more cells in the infarcted region as compared to the other delivery routes. However, the procedural risk for stereotaxic injection inevitably raises the safety concern. Some investigators reported that using intraparenchymal cell implantation exhibits severe adverse events in early clinical trials, involving motor worsening, seizures, syncope, and chronic subdural hematoma [40]. Recently, intranasal delivery has emerged as a novel strategy of bypassing the BBB to deliver therapeutic agents to the brain. This non-invasive method facilitates cell homing towards the central nervous system and reduces the potential side-effects associated with intravascular administration [41]. Thus, MSCs and NSCs gained access to the brain via the nasal cavity and rendered therapeutic benefits in Parkinson's disease, malignant gliomas, and stroke [42]. Thus, an optimal delivery route for transplanted cells with respect to the migration and differentiation of NSCs and the extent of neurological improvement in cerebral ischemia necessitate further investigation.

Transplanted human NSCs have been shown to differentiate into astrocytes and neurons in the adult rat brain after ischemic stroke. In this study, we identified the differentiation potential of the transplanted NSCs in rat brains injured with MCAO. The grafted NSCs expressed the proteins specific for neurons and astrocytes, especially the CMV-bFGF C17.2 stem cells.

## MATERIALS AND METHODS

### Construction of recombinant lentiviral vector containing the *bFGF* gene

Lentiviral-based expression vectors, LV-GFP and LV-bFGF, were constructed using the Gateway system (Invitrogen, USA). Full-length bFGF, CMV promoter, and GFP sequences were cloned into plasmids. The vectors were obtained with incubation of donor and accepter vectors catalyzed by LR clonase (Gate-way® LR Clonase^TM^ Plus Enzyme Mix, Invitrogen). Subsequently, the plasmids were sequenced to confirm the inserts. 293T cells were transfected with the constructs and packaging plasmids using Lipofectamine 2000 (Invitrogen, USA). After 3 days, the supernatants of the cells were collected and the viral particles harvested by ultracentrifugation at 100,000 ×*g* for 1 h and resuspended in Dulbecco-modified Eagle's medium (DMEM) for transduction.

### Lentiviral transduction

The C17.2 cells were seeded at a density of 2 × 10^5^ cells/well in 24-well plate and exposed to the viral particles in 0.5 mL DMEM at 37°C for 4 h. Then, the cells were transduced with LV-GFP and LV-bFGF using polybrene (a final concentration of 8 μg/mL). Subsequently, the medium was removed, and the cells were washed once with DMEM and re-cultured in normal medium with blasticidin (2 μg/mL) for 14 days. The untransduced cells were eliminated after culturing for 14 days cultured in medium with blasticidin. Following stable selection, we found that GFP signal initially observed after transduction was barely detected by confocal analysis, suggesting that IRESs in these constructs were not active in the C17.2 cells under the experimental conditions described above. However, the bFGF expression was confirmed by Western blot.

### Western blot analysis

The cells were lysed in RIPA buffer and incubated for 10 min on ice and centrifuged at 12,000 rpm for 10 min at 4°C. Then, the whole protein samples were separated on 12% gradient SDS-PAGE and transferred to PVDF membranes (Bio-Rad, Hercules, CA, USA) that were blocked in 5% skim milk for 90 min and probed with the primary antibody anti-bFGF (1:500, sc-79, Santa Cruz Biotechnology, Santa Cruz, CA, USA) at 4°C overnight. After washing three times with 0.1% Tween-20 in Tris-buffered saline (TBS), the membrane was incubated with the secondary antibody for 1 h at room temperature. Consequently, the target protein was detected using a ChemiDoc^TM^ XRS+ Imaging System (Bio-Rad).

### Cell culture and preparation

The C17.2 cells were maintained in DMEM supplemented with 10% fetal bovine serum (Gibco, NY, USA), 5% horse serum (Gibco, NY, USA), 2 mM glutamine, and penicillin/streptomycin (Gibco, NY, USA). The cells were cultured in a humidified atmosphere of 5% CO_2_ and 95% air at 37°C and routinely split at approximately 90% confluency [43].

For grafted cell identification, NSCs were labeled with Cell Tracker CM-DiI (Invitrogen, USA) before transplantation by incubating the cells with the dye for 5 min at 37°C and an additional 15 min at 4°C. The labeling of the cells was verified to be 99-100%, under a fluorescent microscope, prior to all transplantations. The cell viability was determined by trypan blue staining at the end of the harvest and before infusion; > 95% viability was found for every infusion.

### OGD treatment and cell viability assays

OGD was used to simulate the environment of cerebral ischemia. The cells were seeded at a density of 6000 cells/well in 96-well plate. For OGD treatment, the complete culture medium was removed and replaced with the serum-free medium after cell adherence, incubated in the hypoxic chamber (Thermo Forma Anaerobic System 1029, MA, USA) with < 1% O_2_. After 24 h, cells were harvested, and 20 μL MTT (3-(4,5-dimethylthiazol-2-yl)-2,5-diphenyltetrazolium bromide) was added to each well, and the plates incubated in a CO_2_ incubator for an additional 4 h. Finally, the medium was aspirated, and 150 μL dimethyl sulfoxide (DMSO) was added to each well to solubilize the formazan crystals. The absorbance was measured using a multi-well microplate reader (Thermo, MA, USA) at 490 nm.

### Animal model

All experimental procedures were approved by the Care of Experimental Animals Committee of Wenzhou Medical University. Adult Sprague–Dawley (SD) male rats (270–300 g) were obtained from Shanghai Laboratory Animal Center.

A transient 120 min right MCAO was performed in rats, as described previously [44]. Briefly, rats were anesthetized with 10% chloral hydrate (1 mL/100 g, i.p.). Rectal temperature was maintained at 37°C using a thermistor-controlled heat blanket. The right common carotid artery (CCA), external carotid artery (ECA), and internal carotid artery (ICA) were exposed. A 5-0 monofilament nylon suture, with its tip rounded by heating near a flame, was advanced from the ECA into the lumen of the ICA until a slight resistance was exerted (18.5–19.5 mm). After 120 min of MCAO, blood flow was restored by removal of the sutures. All animals were returned to their cages with free access to food and water under a 12-h light-dark cycle.

### Cells transplantation and BrdU labeling

The experiments were divided into 3 groups, including PBS, CMV-hrGFP C17.2, and CMV-bFGF C17.2 groups (16 animals in each group). After 24 h of MCAO, the animals were anesthetized by 10% chloral hydrate solution and underwent transplantation with C17.2 cells or phosphate-buffered saline (PBS). Approximately 5×10^6^ CM-DiI-labeled cells in a volume of 200 μL PBS or an equivalent volume of PBS alone were injected into the rat tail vein. Then, 5 randomly selected experimental animals from each group were injected with 5-bromo-2’-deoxyuridine (50 mg/kg BrdU, Sigma, USA) on the day of transplantation and consecutive for 28 days. The animals were executed on day 28 day after the final BrdU injection.

### Behavioral tests

The behavioral tests were performed before MCAO and at 1, 3, 7, 10, 14, 21, and 28 days after intravenous transplantation by an investigator who was blinded to the experimental groups, as described previously [45]. The neurological severity score (NSS) consists of 5 raising the tail tests, 4 placed on the floor tests, 3 sensory tests and coordination, and balance behavior including 3 beam tests. The neurological functioning was rated on NSS scale of 0 to 18 points (normal score: 0, maximal score: 18), where 0 indicated a normal repair effect and 18 suggested total impairment. Thus, the higher the score the severe the injury.

### TTC staining and quantitative analysis of infarct volume

One week post-transplantation, 5 rats in each group were anesthetized with 10% chloral hydrate. The brains were removed carefully and dissected into 2-mm thick coronal sections. The fresh brain slices were immersed in 0.5% solution of TTC (Sigma, USA) in PBS at 37°C for 30 min. The cross-sectional area of the infarction in each brain slice was calculated by Image J analysis software (NIH, USA). The total infarct volume for each brain was calculated by the summation of the infarcted area of all brain slices.

### Immunofluorescence analysis

At 28 days after MCAO, the rats were euthanized by 10% chloral hydrate. The brains were perfused and fixed with 4% paraformaldehyde for 24 h, dehydrated by increasing concentrations of saccharose, embedded in OCT (optimal cutting temperature compound, Sakura, USA), cut into 5-μm thick sections in the coronal plane. For endogenous proliferation, the cells were stained with the primary antibody anti-BrdU (1:1000; ab8152, Abcam, UK) at 28 days after MCAO. Subsequently, the slides were stained with the following primary antibodies: a basic fibroblast growth factor antigen, bFGF (1:200, sc-79, Santa Cruz Biotechnology, Santa Cruz, CA, USA); a neuronal nuclear antigen, NeuN (1:1000, ab104225, Abcam, UK), an astrocytic marker, GFAP (1:200, sc-6170, Santa Cruz Biotechnology, USA), and a neural stem cell marker, Nestin (1:1000, ab6142, Abcam, UK). The negative control slides for each animal underwent identical preparations for immunohistochemical staining, except the treatment with the primary antibodies.

### Statistical analysis

Data are expressed as the mean ± SEM. Statistical significance was determined by Student's t-test in the case of two experimental groups. For more than two groups, the data were statistically evaluated using one-way analysis of variance (ANOVA) followed by Dunnett's post hoc test. For all tests, a value of *P* ≤ 0.05 was considered as statistically significant.

## CONCLUSION

In summary, the transplantation of transgenic NSCs, especially bFGF modification, may provide a valuable tool for improving the efficiency of the treatment of cerebral ischemia. Therefore, cell transplantation, as a new therapeutic modality, in highly promising for the treatment of stroke and can markedly improve the quality of life of the elderly patients.

## References

[1] Alluri H, Anasooya Shaji C, Davis ML, Tharakan B (2015). Oxygen-glucose deprivation and reoxygenation as an *in vitro* ischemia-reperfusion injury model for studying blood-brain barrier dysfunction. Journal of visualized experiments: JoVE.

[2] Grossman AW, Broderick JP (2013). Advances and challenges in treatment and prevention of ischemic stroke. Annals of neurology.

[3] Hacke W, Kaste M, Bluhmki E, Brozman M, Davalos A, Guidetti D, Larrue V, Lees KR, Medeghri Z, Machnig T, Schneider D, von Kummer R, Wahlgren N (2008). Thrombolysis with alteplase 3 to 4.5 hours after acute ischemic stroke. The New England journal of medicine.

[4] Liu X, Ye R, Yan T, Yu SP, Wei L, Xu G, Fan X, Jiang Y, Stetler RA, Liu G, Chen J (2014). Cell based therapies for ischemic stroke: from basic science to bedside. Progress in neurobiology.

[5] Kalluri HS, Dempsey RJ (2008). Growth factors, stem cells, and stroke. Neurosurgical focus.

[6] Zhang P, Li J, Liu Y, Chen X, Kang Q, Zhao J, Li W (2009). Human neural stem cell transplantation attenuates apoptosis and improves neurological functions after cerebral ischemia in rats. Acta anaesthesiologica Scandinavica.

[7] Kelly S, Bliss TM, Shah AK, Sun GH, Ma M, Foo WC, Masel J, Yenari MA, Weissman IL, Uchida N, Palmer T, Steinberg GK (2004). Transplanted human fetal neural stem cells survive, migrate, and differentiate in ischemic rat cerebral cortex.

[8] Szentirmai O, Carter BS (2004). Genetic and cellular therapies for cerebral infarction. Neurosurgery.

[9] Hicks AU, Lappalainen RS, Narkilahti S, Suuronen R, Corbett D, Sivenius J, Hovatta O, Jolkkonen J (2009). Transplantation of human embryonic stem cell-derived neural precursor cells and enriched environment after cortical stroke in rats: cell survival and functional recovery. The European journal of neuroscience.

[10] Okada-Ban M, Thiery JP, Jouanneau J (2000). Fibroblast growth factor-2. The international journal of biochemistry & cell biology.

[11] Wada K, Sugimori H, Bhide PG, Moskowitz MA, Finklestein SP (2003). Effect of basic fibroblast growth factor treatment on brain progenitor cells after permanent focal ischemia in rats. Stroke.

[12] Ren JM, Finklestein SP (2005). Growth factor treatment of stroke. Current drug targets CNS and neurological disorders.

[13] Zhao YZ, Lin M, Lin Q, Yang W, Yu XC, Tian FR, Mao KL, Yang JJ, Lu CT, Wong HL (2016). Intranasal delivery of bFGF with nanoliposomes enhances *in vivo* neuroprotection and neural injury recovery in a rodent stroke model. Journal of controlled release.

[14] Wang ZG, Cheng Y, Yu XC, Ye LB, Xia QH, Johnson NR, Wei X, Chen DQ, Cao G, Fu XB, Li XK, Zhang HY, Xiao J (2016). bFGF Protects Against Blood-Brain Barrier Damage Through Junction Protein Regulation via PI3K-Akt-Rac1 Pathway Following Traumatic Brain Injury. Molecular neurobiology.

[15] Issa R, AlQteishat A, Mitsios N, Saka M, Krupinski J, Tarkowski E, Gaffney J, Slevin M, Kumar S, Kumar P (2005). Expression of basic fibroblast growth factor mRNA and protein in the human brain following ischaemic stroke. Angiogenesis.

[16] van Velthoven CT, Sheldon RA, Kavelaars A, Derugin N, Vexler ZS, Willemen HL, Maas M, Heijnen CJ, Ferriero DM (2013). Mesenchymal stem cell transplantation attenuates brain injury after neonatal stroke. Stroke.

[17] Chang DJ, Lee N, Choi C, Jeon I, Oh SH, Shin DA, Hwang TS, Lee HJ, Kim SU, Moon H, Hong KS, Kang KS, Song J (2013). Therapeutic effect of BDNF-overexpressing human neural stem cells (HB1.F3.BDNF) in a rodent model of middle cerebral artery occlusion. Cell transplantation.

[18] Wolf WA, Martin JL, Kartje GL, Farrer RG (2014). Evidence for fibroblast growth factor-2 as a mediator of amphetamine-enhanced motor improvement following stroke. PloS one.

[19] Ay H, Ay I, Koroshetz WJ, Finklestein SP (1999). Potential usefulness of basic fibroblast growth factor as a treatment for stroke. Cerebrovascular diseases.

[20] Gladstone DJ, Black SE, Hakim AM (2002). Heart and Stroke Foundation of Ontario Centre of Excellence in Stroke Recovery. Toward wisdom from failure: lessons from neuroprotective stroke trials and new therapeutic directions. Stroke.

[21] Ye LB, Yu XC, Xia QH, Yang Y, Chen DQ, Wu F, Wei XJ, Zhang X, Zheng BB, Fu XB, Xu HZ, Li XK, Xiao J, Zhang HY (2017). Erratum to: Regulation of Caveolin-1 and Junction Proteins by bFGF Contributes to the Integrity of Blood-Spinal Cord Barrier and Functional Recovery. Neurotherapeutics.

[22] Schaar KL, Brenneman MM, Savitz SI (2010). Functional assessments in the rodent stroke model. Experimental & translational stroke medicine.

[23] De Feo D, Merlini A, Laterza C, Martino G (2012). Neural stem cell transplantation in central nervous system disorders: from cell replacement to neuroprotection. Current opinion in neurology.

[24] Bourzac K (2016). Neuroscience: New nerves for old. Nature.

[25] Guzman R, De Los Angeles A, Cheshier S, Choi R, Hoang S, Liauw J, Schaar B, Steinberg G (2008). Intracarotid injection of fluorescence activated cell-sorted CD49d-positive neural stem cells improves targeted cell delivery and behavior after stroke in a mouse stroke model. Stroke.

[26] Vergano-Vera E, Mendez-Gomez HR, Hurtado-Chong A, Cigudosa JC, Vicario-Abejon C (2009). Fibroblast growth factor-2 increases the expression of neurogenic genes and promotes the migration and differentiation of neurons derived from transplanted neural stem/progenitor cells. Neuroscience.

[27] Jin-qiao S, Bin S, Wen-hao Z, Yi Y (2009). Basic fibroblast growth factor stimulates the proliferation and differentiation of neural stem cells in neonatal rats after ischemic brain injury. Brain & development.

[28] Smith HK, Gavins FN (2012). The potential of stem cell therapy for stroke: is PISCES the sign?. FASEB journal.

[29] Shen CC, Lin CH, Yang YC, Chiao MT, Cheng WY, Ko JL (2010). Intravenous implanted neural stem cells migrate to injury site, reduce infarct volume, and improve behavior after cerebral ischemia. Current neurovascular research.

[30] Ikegame Y, Yamashita K, Hayashi S, Mizuno H, Tawada M, You F, Yamada K, Tanaka Y, Egashira Y, Nakashima S, Yoshimura S, Iwama T (2011). Comparison of mesenchymal stem cells from adipose tissue and bone marrow for ischemic stroke therapy. Cytotherapy.

[31] Wurmser AE, Nakashima K, Summers RG, Toni N, D'Amour KA, Lie DC, Gage FH (2004). Cell fusion-independent differentiation of neural stem cells to the endothelial lineage. Nature.

[32] Andsberg G, Kokaia Z, Bjorklund A, Lindvall O, Martinez-Serrano A (1998). Amelioration of ischaemia-induced neuronal death in the rat striatum by NGF-secreting neural stem cells. The European journal of neuroscience.

[33] Yang M, Stull ND, Berk MA, Snyder EY, Iacovitti L (2002). Neural stem cells spontaneously express dopaminergic traits after transplantation into the intact or 6-hydroxydopamine-lesioned rat. Experimental neurology.

[34] Ryder EF, Snyder EY, Cepko CL (1990). Establishment and characterization of multipotent neural cell lines using retrovirus vector-mediated oncogene transfer. Journal of neurobiology.

[35] Zhu JM, Zhao YY, Chen SD, Zhang WH, Lou L, Jin X (2011). Functional recovery after transplantation of neural stem cells modified by brain-derived neurotrophic factor in rats with cerebral ischaemia. The Journal of international medical research.

[36] Snyder EY, Deitcher DL, Walsh C, Arnold-Aldea S, Hartwieg EA, Cepko CL (1992). Multipotent neural cell lines can engraft and participate in development of mouse cerebellum. Cell.

[37] Misra V, Ritchie MM, Stone LL, Low WC, Janardhan V (2012). Stem cell therapy in ischemic stroke: role of IV and intra-arterial therapy. Neurology.

[38] Zhang L, Li Y, Romanko M, Kramer BC, Gosiewska A, Chopp M, Hong K (2012). Different routes of administration of human umbilical tissue-derived cells improve functional recovery in the rat after focal cerebral ischemia. Brain research.

[39] Li L, Jiang Q, Ding G, Zhang L, Zhang ZG, Li Q, Panda S, Lu M, Ewing JR, Chopp M (2010). Effects of administration route on migration and distribution of neural progenitor cells transplanted into rats with focal cerebral ischemia, an MRI study. Journal of cerebral blood flow and metabolism.

[40] Lim JY, Jeong CH, Jun JA, Kim SM, Ryu CH, Hou Y, Oh W, Chang JW, Jeun SS (2011). Therapeutic effects of human umbilical cord blood-derived mesenchymal stem cells after intrathecal administration by lumbar puncture in a rat model of cerebral ischemia. Stem cell research & therapy.

[41] Wei N, Yu SP, Gu X, Taylor TM, Song D, Liu XF, Wei L (2013). Delayed intranasal delivery of hypoxic-preconditioned bone marrow mesenchymal stem cells enhanced cell homing and therapeutic benefits after ischemic stroke in mice. Cell transplantation.

[42] Danielyan L, Schafer R, von Ameln-Mayerhofer A, Bernhard F, Verleysdonk S, Buadze M, Lourhmati A, Klopfer T, Schaumann F, Schmid B, Koehle C, Proksch B, Weissert R (2011). Therapeutic efficacy of intranasally delivered mesenchymal stem cells in a rat model of Parkinson disease. Rejuvenation research.

[43] Doering LC, Snyder EY (2000). Cholinergic expression by a neural stem cell line grafted to the adult medial septum/diagonal band complex. Journal of neuroscience research.

[44] Belayev L, Alonso OF, Busto R, Zhao W, Ginsberg MD (1996). Middle cerebral artery occlusion in the rat by intraluminal suture. Neurological and pathological evaluation of an improved model. Stroke.

[45] Chen J, Li Y, Chopp M (2000). Intracerebral transplantation of bone marrow with BDNF after MCAo in rat. Neuropharmacology.

